# Evaluation of lymphocyte apoptosis in patients with oral cancer

**DOI:** 10.1590/1678-7757-2020-0124

**Published:** 2020-09-07

**Authors:** Fardeela BIN-ALEE, Areeya ARAYATAWEEGOOL, Supranee BURANAPRADITKUN, Patnarin MAHATTANASAKUL, Napadon TANGJATURONRASME, Apiwat MUTIRANGURA, Nakarin KITKUMTHORN

**Affiliations:** 1 Chulalongkorn University Faculty of Medicine Department of Anatomy Bangkok Thailand Chulalongkorn University, Faculty of Medicine, Department of Anatomy, Center of Excellence in Molecular Genetics of Cancer and Human Diseases, Bangkok, Thailand.; 2 Chulalongkorn University Faculty of Medicine Program of Medical Science Bangkok Thailand Chulalongkorn University, Faculty of Medicine, Program of Medical Science, Bangkok, Thailand.; 3 Chulalongkorn University Faculty of Medicine Department of Medicine Bangkok Thailand Chulalongkorn University, Faculty of Medicine, Department of Medicine, Division of Allergy and Clinical Immunology, Bangkok, Thailand.; 4 King Chulalongkorn Memorial Hospital Bangkok Thailand King Chulalongkorn Memorial Hospital, Bangkok, Thailand.; 5 Chulalongkorn University Faculty of Medicine Center of Excellence in Vaccine Research and Development Bangkok Thailand Chulalongkorn University, Faculty of Medicine, Center of Excellence in Vaccine Research and Development (Chula Vaccine Research Center- Chula VRC), Bangkok, Thailand.; 6 Thai Red Cross Society King Chulalongkorn Memorial Hospital Department of Otolaryngology, Head and Neck Surgery Bangkok Thailand Thai Red Cross Society, King Chulalongkorn Memorial Hospital, Department of Otolaryngology, Head and Neck Surgery, Bangkok, Thailand.; 7 Chulalongkorn University Faculty of Medicine Department of Otolaryngology, Head and Neck Surgery Bangkok Thailand Chulalongkorn University, Faculty of Medicine, Department of Otolaryngology, Head and Neck Surgery, Bangkok, Thailand.; 8 Mahidol University Faculty of Dentistry Department of Oral Biology Bangkok Thailand Mahidol University, Faculty of Dentistry, Department of Oral Biology, Bangkok, Thailand.

**Keywords:** Apoptosis, Bax/Bcl-2, Lymphocyte, Oral cancer, Neoplasm staging

## Abstract

**Objectives:**

To evaluate apoptotic levels of peripheral blood mononuclear cells (PBMCs) and apoptotic regulatory proteins (Bax and Bcl-2) in lymphocyte subsets of oral cancer (OC) patients and healthy controls (HC).

**Methodology:**

The percentage of apoptotic cells and lymphocyte counts were measured in the first cohort using PBMCs obtained from 23 OC patients and 6 HC. In the second cohort, (OC, 33; HC, 13), the mean fluorescence intensity (MFI) of Bax and Bcl-2 in CD19^+^ B, CD4^+^ T, CD8^+^ T, and CD16^+^56^+^ natural killer (NK) cells was determined via flow cytometry.

**Results:**

The percentage of apoptotic cells was higher in the PBMCs of OC patients than in HC patients, particularly in patients with stage IV cancer (*p*<0.05). However, lymphocyte counts were significantly lower in stage IV patients (*p*<0.05). NK CD19^+^ B and CD16^+^56^+^ cell counts were significantly lower in OC patients compared with HC patients (*p*<0.001 and *p*<0.01, respectively), but CD4^+^ T cells were interestingly significantly higher in OC patients (*p*<0.001). While Bax MFI was slightly higher, Bcl-2 MFI was significantly lower for all four lymphocyte subsets in OC samples, particularly in stage IV patients, when compared with HC. Consequently, Bax/Bcl-2 ratios showed an upward trend from HC to OC patients, particularly those in stage IV. We found similar trends in Bax and Bcl-2 MFI for tumor stage, tumor size, and lymph node involvement.

**Conclusions:**

The increased lymphocyte apoptosis in stage IV OC patients may be related to higher Bax levels and lower Bcl-2 levels. The Bax/Bcl-2 ratio in lymphocytes may be useful to determine the prognosis of OC patients, and could be considered a mean for supportive treatment in the future.

## Introduction

Oral cancer (OC) is a worldwide common type of malignancy.^1^ This cancer originates from the stratified squamous epithelium of the oral cavity and is called oral squamous cell carcinoma (OSCC).^2^ It is often diagnosed at advanced stages, entailing fatalities and low survival rates.^3^ During metastasis (stage IV), OSCC patients experience weakness and fatigue, are susceptible to infection, and commonly present leukopenia.^4-6^ Low lymphocyte counts (lymphopenia) may lead to a decrease in immune system’s ability to inhibit cancer development, promoting tumor growth and worsening prognosis.^7^

OC has an immune-escape mechanism. Malignant cells are associated with immune suppression, enabling cancer cells to evade the host’s immune surveillance.^7,8^ The impaired function of the immune system might be directly associated with head and neck squamous cell carcinomas (HNSCC) growth and metastasis.^8^ It has also been reported that tumor cells can escape immune surveillance, inhibit immune function,^9^ and induce immunogenic cell death and lymphocyte apoptosis, changing lymphocyte homeostasis.^7^

Apoptosis is a form of programmed cell death that plays a critical role in normal development and homeostasis of adult tissues, including cell turnover, immune system development, embryonic development, and chemical-induced cell death.^10,11^ Bcl-2-associated X (Bax), a pro-apoptotic protein, and B-cell lymphoma-2 (Bcl-2), an anti-apoptotic protein, are interrelated members of the Bcl-2 family proteins, associated with mechanisms that regulate the permeabilization of the mitochondrial outer membrane, a critical step of apoptosis.^12^ Defects in mechanisms of apoptosis are involved in tumor pathogenesis. Tumor cells can acquire resistance to apoptosis by Bax downregulation or mutation and Bcl-2 upregulation. Bcl-2 and Bax expression is regulated by p53, a tumor suppressor gene.^13^ A previous study on cancer tissues found that the expression of Bax is strongly correlated with good clinical outcomes in HNSCC patients.^14^ Takemura and Noguchi^15^ (2002) corroborate with these results, reporting that patients with OSCC along with Bax expression had better prognosis than those without Bax expression. Some studies demonstrated that lower levels of Bcl-2 and higher levels of Bax are associated with overall clinical improvement in patients with OSCC.^16,17^

Under normal circumstances, apoptosis plays a key role within the immune system. A previous study observed lower levels of Bcl-2 in T cells of patients with HNSCC in comparison to healthy donors.^18^ Several studies, conducted in various conditions, reported that Bcl-2 and Bax are crucial for the survival and proliferation of several types of cells, such as CD4^+^ T, B, and natural killer (NK) cells.^18-20^

Our study focused on lymphocyte apoptosis, hypothesizing that lymphopenia in OC patients is associated with lymphocyte apoptosis. For that, we measured the levels of Bax and Bcl-2 in common lymphocyte subsets – CD19^+^ B, CD4^+^ T, CD8^+^ T, and CD16^+^56^+^ NK cells – for both OC patients and healthy controls (HC).

## Methodology

### Ethical statement

This study was approved by the Institutional Review Board of the Faculty of Medicine of Chulalongkorn University (IRB No. 228/63), and was conducted according to the ethical principles established by the Declaration of Helsinki . All participants agreed to participate by signing a consent form before the start of the study.

### Samples

In the first cohort, peripheral blood mononuclear cells (PBMCs) were collected for detecting apoptosis. In total, 25 samples were obtained from OC patients (stage I: 4; stage II: 1; stage III: 4; and stage IV: 16) and 6 from HC (Table 1). In the second cohort, the levels of Bax and Bcl-2 apoptotic regulatory proteins were measured in 33 OC samples (stage I: 6; stage II: 3; stage III: 5; and stage IV: 19) and 13 HC samples (Table 1). All samples were collected from the Department of Otolaryngology, Head and Neck Surgery, Faculty of Medicine, Chulalongkorn University, Thailand. OSCC was confirmed via histopathological analysis, conducted by a pathologist (NK), and clinical staging was recorded using the tumor, node, and metastasis (TNM) staging system (PM, NT, and VK). PBMCs were isolated from heparinized blood using Ficoll–Paque density gradient centrifugation, following manufacturer’s instructions (Axis-Shield PoC AS, Oslo, Norway).

**Table 1:** Detailed data of samples in cohorts 1 and 2

Experiment	Code	Sex	Age	Staging	Tumor size	Lymph node involvement	Metastasis
	HC1	F	58	-	-	-	-
	HC2	M	68	-	-	-	-
	HC3	F	55	-	-	-	-
	HC4	F	58	-	-	-	-
	HC5	F	58	-	-	-	-
	HC6	M	56	-	-	-	-
	OC1	M	64	I	T1	N0	M0
	OC2	M	57	I	T1	N0	M0
	OC3	M	33	I	T1	N0	M0
	OC4	M	78	I	T1	N0	M0
	OC5	F	43	II	T2a	Nx	Mx
	OC6	M	70	III	T3	N1	M0
	OC7	M	61	III	T1	N1	M0
	OC8	M	50	III	T3	N0	M0
**Percentage of lymphocytes and apoptotic cells**	OC9	M	68	III	T3	N1	M0
	OC10	F	43	IV	T4a	N1	M0
	OC11	M	50	IV	T4a	N2	Mx
	OC12	M	45	IV	T4b	N2	Mo
	OC13	M	45	IV	T4a	N1	Mx
	OC14	F	62	IV	T4a	N0	M0
	OC15	F	26	IV	T4a	N3	Mx
	OC16	F	60	IV	T4a	No	Mx
	OC17	F	62	IV	T4b	N0	Mx
	OC18	F	53	IV	T4a	N0	Mx
	OC19	M	80	IV	T4	N2a	Mx
	OC20	M	54	IV	T3	N2c	Mx
	OC21	M	71	IV	T4b	N0	M0
	OC22	M	41	IV	T2	N2	M0
	OC23	M	76	IV	T4a	N2b	M1
	OC24	M	59	IV	T2	N0	M1
	OC25	M	45	IV	T4a	N2b	M0
	HC7	M	48	-	-	-	-
	HC8	F	52	-	-	-	-
	HC9	F	58	-	-	-	-
	HC10	M	68	-	-	-	-
	HC11	F	55	-	-	-	-
	HC12	F	58	-	-	-	-
	HC13	F	58	-	-	-	-
	HC14	M	56	-	-	-	-
	HC15	M	56	-	-	-	-
	HC16	M	23	-	-	-	-
	HC17	F	48	-	-	-	-
	HC18	M	57	-	-	-	-
	HC19	F	22	-	-	-	-
	OC26	M	65	I	T1	N0	M0
	OC27	M	59	I	T1	N0	M0
	OC28	M	40	I	T1	N0	M0
	OC29	F	61	I	T1	N0	M0
	OC30	M	75	I	T1	N0	M0
	OC31	M	74	I	T1	N0	M0
	OC32	F	45	II	T2a	Nx	Mx
	OC33	F	80	II	T2	N0	M0
	OC34	F	55	II	T2	N0	M0
**MFI of Bax and Bcl-2**	OC35	M	63	III	T3	N1	M0
	OC36	M	61	III	T1	N1	M0
	OC37	M	52	III	T3	N0	M0
	OC38	M	59	III	T3	N1	M0
	OC39	M	65	III	T3	N1	M0
	OC40	F	40	IV	T4a	N1	M0
	OC41	M	58	IV	T4a	N2	Mx
	OC42	M	46	IV	T4b	N2	Mo
	OC43	M	45	IV	T4a	N1	Mx
	OC44	F	61	IV	T4a	N0	M0
	OC45	F	26	IV	T4a	N3	Mx
	OC46	F	54	IV	T4a	No	Mx
	OC47	F	63	IV	T4b	N0	Mx
	OC48	F	59	IV	T4a	N0	Mx
	OC49	M	74	IV	T4	N2a	Mx
**MFI of Bax and Bcl-2**	OC50	M	58	IV	T3	N2c	Mx
	OC51	M	70	IV	T4b	N0	M0
	OC52	M	46	IV	T2	N2	M0
	OC53	M	42	IV	T4a	N2	M0
	OC54	M	40	IV	T3	N2	M0
	OC55	F	54	IV	T4a	N2b	M0
	OC56	M	76	IV	T4a	N2b	M1
	OC57	M	60	IV	T2	N0	M1
	OC58	M	47	IV	T4a	N2b	M0

F = female, HC = healthy control, M = male, OC = oral cancer patient

### Apoptosis of PBMCs and lymphocyte count

PBMCs and gated lymphocytes were used to analyze data on apoptosis. The first cohort investigated lymphocyte count and the presence of apoptosis in samples from OC patients and HC. One million cells were stained with Annexin V Alexa Fluor 488 and propidium iodide (Biolegend, San Diego, CA, USA) for 30 min at room temperature. Fluorescence-activated cell sorting (FACS) buffer was added to the cells for quantifying cell types via flow cytometry (LSRII, BD biosciences, CA, USA). Frequencies of lymphocytes were gated from forward scatter FCS and side scatter (SSC). Data were analyzed using the FlowJo program (Ashland, OR, USA).

### Immune cell subsets with Bax and Bcl-2 measurements

**Figure 1:**
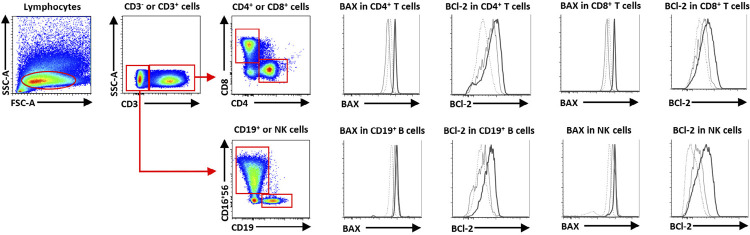
Gating strategy of PBMCs via flow cytometry. Lymphocytes were gated from FSC and SSC based on size and granularity. CD3 was used to identify T cells (CD3+) or non-T cells (CD3-). Staining with CD4 and CD8 antibodies against CD4 and CD8 T cells from CD3+ T cells and CD19 Ab for B cells and CD16/56 Ab for NK cells from CD3- T cells. Gated Bax and Bcl-2 expression of immune cells represented by mean fluorescence intensity (MFI). Bax and Bcl-2 expression in HC (dash line), low-expression OC (black line), and high-expression OC (solid black line)

The second cohort categorized PBMCs into four types of immune cells based on cell-surface markers, as follows: CD19^+^ B, CD4^+^ T, CD8^+^ T, and CD16^+^CD56^+^ NK. The mean fluorescence intensities (MFI) of Bax and Bcl-2 were measured within these lymphocyte subsets. One million PBMCs were washed with phosphate-buffered saline with 2% fetal bovine serum (FBS; FACS buffer) and stained with cell-surface markers for 20 min at 4°C. The cell-surface markers contained PE-DZ594-labeled anti-CD16 (clone 3G8) and anti-CD56 (clone 5.1H11), PerCP-Cy5.5-labeled anti-CD19 (clone HIB19), PE-Cy7-labeled anti-CD3 (clone UCHT1), Alexa Fluor 700-labeled anti-CD8 (clone SK1), and APC-Cy7-labeled anti-CD4 (clone RPA-T4) (Biolegend, San Diego, CA, USA). After washing the cells twice in FACS buffer, they were fixed/permeabilized and stained in Alexa Fluor 488 anti-Bax (clone 2D2) and PE anti-Bcl-2 (clone 100) (Biolegend, San Diego, CA, USA) antibodies for 30 min at 4°C. Then, cells were again washed in FACS buffer, fixed in 2% paraformaldehyde (2% PFA), and analyzed via flow cytometry. Figure 1 shows an example of the gating strategies used in flow cytometry for detecting Bax and Bcl-2 in CD19+ B, CD4+ T, CD8+ T, and CD16+56+ NK cells among HC (dash line), low-expression OC (black line), and high-expression OC (solid black line).

### Statistical analysis

All statistical analyses were performed using SPSS v. 22 (SPSS Inc., Chicago, IL, USA). One-way analysis of variance (ANOVA) and unpaired t-test calculated significant differences in the first cohort. Kruskal–Wallis test compared MFI among the groups in the second cohort. Pearson’s correlation coefficient (R) was used to assess correlations between apoptosis and different cell counts for each group. A *p*-value of less than 0.05 (<0.05) was considered statically significant.

## Results

### Percentage of lymphocytes and apoptotic PBMCs between OC patients and HC

**Figure 2:**
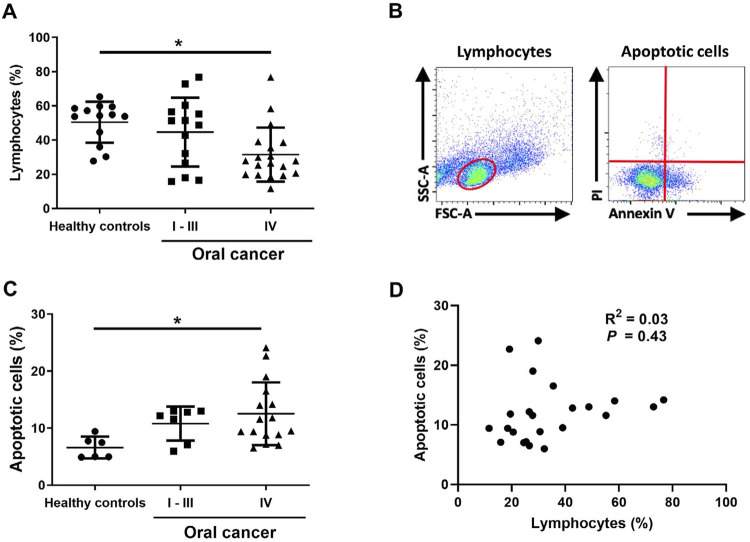
Percentage of lymphocytes. (A), Gated lymphocytes (B) and apoptotic cells (C). Correlations observed between the percentage of lymphocytes and apoptotic cells in OC cells (D) among the PBMCs obtained from the OC patients and HC. *: *p*<0.05

Given the small sample of OC patients in the stages I, II, and III, we merged them into one group (OC stage I–III). The percentage of lymphocytes was higher in HC group, followed by OC stage I–III, and OC stage IV. The percentage of lymphocytes was significantly lower in OC stage IV than in HC samples (*p*<0.05; Figure 2A). However, the percentage of apoptotic cells gated from lymphocytes (Figure 2B) gradually increased from HC, to OC stage I–III, and OC stage IV. We found a significant difference between patients with OC stage IV and HC (*p*<0.05; Figure 2C), but no significant correlations between the percentage of lymphocytes and apoptotic cells within OC samples (R^2^=0.03; *p*=0.43; Figure 2D).

### Percentage of lymphocyte subtypes and Bax/Bcl-2 MFI and ratio between OC patients and HC

**Figure 3:**
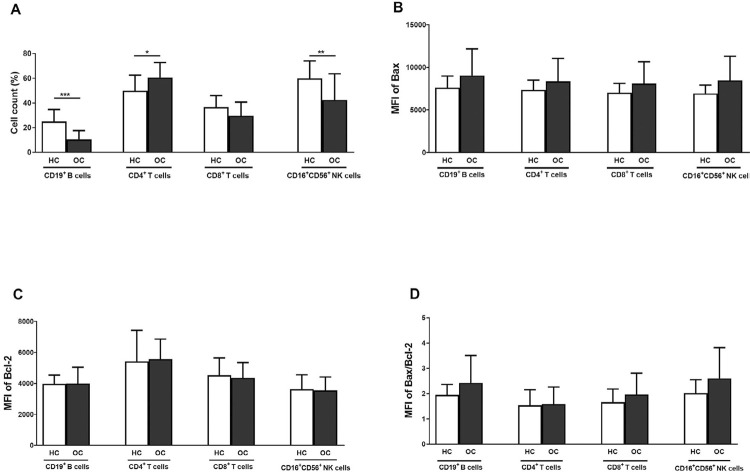
Comparison between OC and HC samples. Frequency lymphocyte subsets (A) and MFI of Bax (B), Bcl-2**: P<0.01 (C), and Bax/Bcl-2 ratio (D) of the four lymphocyte cell types. *: *p*<0.05, **: *p*<0.01, ***: *p*<0.001

Figure 3A shows lymphocyte subsets. OC samples presented significantly higher CD4^+^ T cells (*p*<0.001) and significantly lower CD8^+^ T cells than HC. CD19^+^ B cells and CD16^+^56^+^ NK cells were significantly lower in OC than in HC samples (*p*<0.001 and *p*<0.01, respectively). Bax MFI of all four cell types was slightly higher in OC samples than in HC samples (Figure 3B). The two groups had similar Bcl-2 MFI levels (Figure 3C). Bax/Bcl-2 ratio in CD19^+^ B, CD8^+^ T, and CD16^+^56^+^ NK cells were slightly higher in OC samples than in HC samples. The mean ± SD of OC *vs.* HC in CD19^+^ B cells were 2.42±1.08 *vs.* 1.95±0.41 (*p*=0.13) , in CD8^+^ T cells 1.96±0.85 *vs.* 1.66±0.52 (*p*=0.24), and in CD16^+^56^+^ NK cells 2.60±1.21 *vs*. 2.02±0.53 (*p*=0.11) (Figure 3D).

### Percentage of lymphocyte subtypes and Bax/Bcl-2 MFI and ratio between OC patients in stage I–III and stage IV

**Figure 4:**
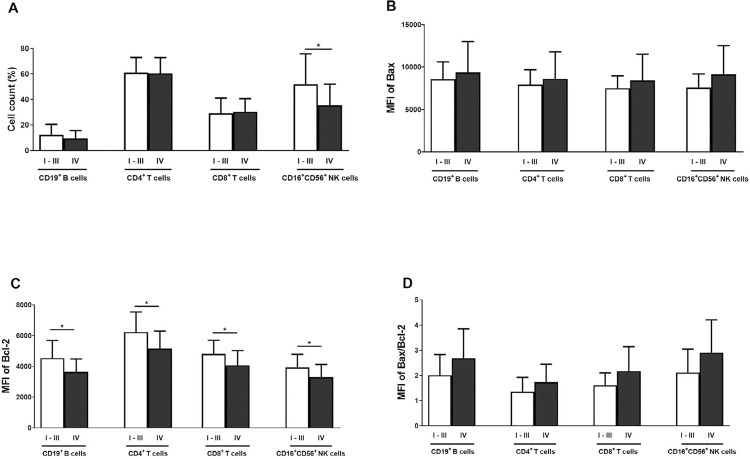
Comparison between stage I–III and stage IV OC samples. Frequency of lymphocyte subsets (A) and MFI of Bax (B), Bcl-2 (C), and Bax/Bcl-2 ratio (D) of the four lymphocyte cell types. *: *p*<0.05

The percentage of CD16^+^56^+^ NK cells was significantly lower in OC stage IV than in stage I–III (*p*<0.05; Figure 4A). Bax MFI of all four cell types was slightly higher in stage IV than in stage I–III samples (Figure 4B). Conversely, Bcl-2 MFI of all four cell types was significantly lower in stage IV (*p*<0.05; Figure 4C). Bax/Bcl-2 ratio in CD19^+^ B, CD4^+^ T, CD8^+^ T, and CD16^+^56^+^ NK cells had slightly higher levels in OC stage IV than in stage I-III samples. The mean ± SD of OC *vs.* HC in CD19^+^ B cells were 2.69±1.16 *vs.* 2.01±0.82 (*p*=0.09), in CD4^+^ T 1.74±0.71 *vs*. 1.36 ±0.56 (*p*=0.13), in CD8+ T 2.18±0.96 *vs*. 1.62±0.49 (*p*=0.07), and in CD16^+^56^+^ NK cells 2.91±1.30 *vs*. 2.12±0.92 (*p*=0.08) Figure 4D).

### Percentage of lymphocytes subtypes and Bax/Bcl-2 MFI and ratio according to tumor size and lymph node metastasis

**Figure 5:**
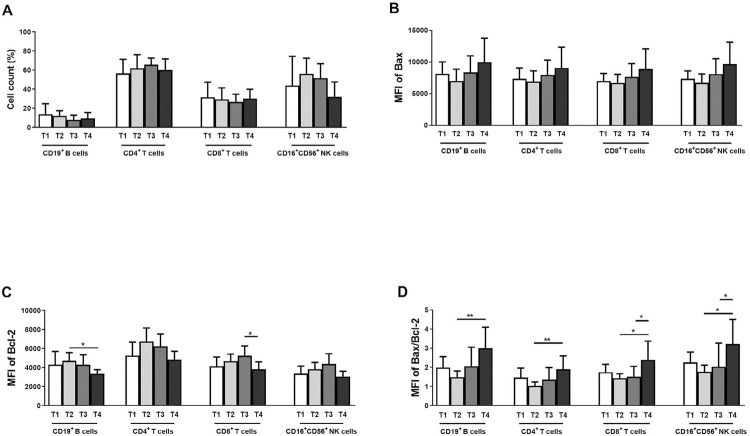
Comparison based on tumor size among T1, T2, T3, and T4 samples. Percentage of cells in the four lymphocyte subtypes (A). MFI of Bax, Bcl-2, and Bax/Bcl-2 of the four lymphocyte cell types (B-D). *: *p*<0.05, **: *p*<0.01

Regarding tumor size, the percentage of CD16^+^CD56^+^ NK cells was significantly lower in T4 than in T1, T2, and T3 of OC samples (Figure 5A). Bax MFI of all four cell types gradually increased from T2 to T3 and T4 (Figure 5B). Bcl-2 MFI of all four cell types – particularly CD19^+^ B cells and CD8^+^ T cells – was considerably lower in T4 tumors (Figure 5C). Bax/Bcl-2 ratio was significantly higher in T4 than in T2 tumors for all four cell types, mainly for CD19^+^ B cells and CD4^+^ T cells (*p*<0.01) and CD8^+^ T and CD16^+^56^+^ NK cells (*p*<0.05) (Figure 5D). Likewise, Bax/Bcl-2 ratio for CD8^+^ T and CD16^+^56^+^ NK cells was significantly higher in T4 than in T3 (Figure 5D; *p* < 0.05).

**Figure 6:**
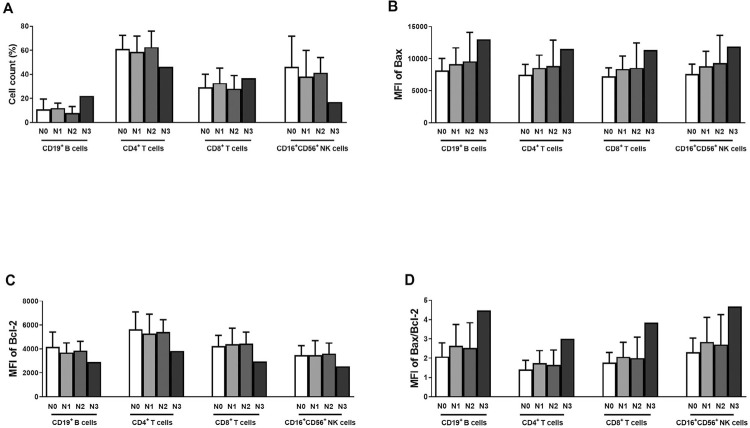
Comparison based on lymph node metastasis among N0, N1, N2, and N3 of samples. Percentage of cells in the four lymphocyte subtypes (A). MFI of Bax, Bcl-2, and Bax/Bcl-2 of the lymphocytes cell types (B–D)

As for the number of metastatic lymph nodes, the percentage of CD4^+^ T cells and CD16^+^CD56^+^ NK cells was lower in N3, whereas for CD19^+^ B and CD8^+^ T-cells it was higher (Figure 6A). Both Bax and Bcl-2 MFI were significantly different for all four cell types in N3 when compared with N0, N1, and N2 (Figure 6B, 6C). Bax/Bcl-2 ratio in CD19^+^ B, CD4^+^ T, CD8^+^ T, and CD16^+^56^+^ NK cells had slightly higher levels in N3 than N0, N1, and N2 (data not shown), but without statically significant difference. N3 *p*-value, when compared with N0, N1, and N2 for all four cell types, ranged from 0.48 to 0.99 (Figure 6D).

## Discussion

The immune system is an important defense mechanism to eliminate tumor cells, and the decrease of its functional response is intrinsically associated with cancer’s growth, metastasis, and its recurrence.^21^ OC is an aggressive tumor, particularly in TNM stage IV – which corresponds to the metastatic stage.^3^ Lymphocytes are an important type of immune cells that have been targeted for the treatment of OC^22^ for being capable of recognizing cancer antigen and destroying cancer cells. In a recent study, we found that PBMCs in cancer patients may alter epigenetic regulation and gene expression as an effect of head and neck^23^ and breast cancer.^24^ Previous studies showed that lymphocyte apoptosis is related to weakness and tumor progression.^8,9^ Based on these findings, we hypothesized that cancer cells can alter the levels of proteins associated with lymphocyte function, including apoptosis.

In this study, we found significantly lower CD19^+^ B and CD16^+^56^+^ NK cell counts in OC patients than in HC. The percentage of CD16^+^56^+^ NK cells was significantly lower among OC stage IV patients. Ye, et al.^25^ (2017) reported that circulating tumor cells (often found in advanced-stage cancer) were associated with a decrease in the number of T lymphocyte subsets and NK cells in the peripheral blood of patients with advanced non-small cell lung cancer. As observed in patients with metastatic breast cancer, NK cells activity was also lower in patients with advanced-stage colorectal and prostate cancer.^26^ NK cells are associated with innate immune system and contribute to the first-line defense against cancer and virus infection.^27^ These cells are responsible for producing tumor necrosis factor (TNF), interferon gamma (IFNγ), interleukin-4 (IL-4), and interleukin-13 (IL-13)^28^ and exerting cytolytic activities against tumor cells.^27^ Many reports have shown that NK cells failure is associated with tumor growth.^19^ NK cells role in tumor immune surveillance comprises: inducing ligands activation, decreasing major histocompatibility complex (MHC) class I expression, retargeting via antibody-dependent cell-mediated cytotoxicity, and releasing granzyme, perforin, or cytokines to kill tumor cells.^29^ NK cells have acted as good prognostic markers in OC patients. Our results show that NK cells were reduced in OC patients in stage IV, T4, and N3 (advanced-stage), what suggests that increasing NK cell counts may be a useful alternative-supportive treatment in patients with OSCC.^30,31^

We also found that T helper (Th) cells were significantly higher in OC patients than in HC. This may be explained by the role of Th cells in anti-tumor response in promoting immune response, including the expansion of B cells and cytotoxic T cells, to eliminate tumor cells by secreting cytokines such as TNF and IFNγ.^32^ However, the percentage of Th cells was slightly lower in OC patients with N3 tumors, which may occur because these tumors were highly metastatic and may have escaped the T cell-mediated immune response mechanism by the adaptation of primary tumor antigens.^33^

Several studies on OC demonstrated that circulating peripheral blood lymphocytes, particularly T cells, are significantly lower during tumor progression.^21^ A study conducted by Reichert, et al.^34^ (2002) showed that T cells were present in lower number in the blood circulation and tumor microenvironment due to apoptosis in patients with head and neck cancer, indicating that apoptosis may play a crucial role in the development and progression of some cancers.

The anti-apoptotic Bcl-2 protein and pro-apoptotic Bax protein are involved in the intrinsic apoptosis pathway and respond to cellular stresses, such as DNA damage, γ-irradiation, and oncogene activation.^35^ During normal cell growth, Bax and Bcl-2 levels are balanced. Many studies show that an imbalance between Bax and Bcl-2, with increased levels of Bax and decreased levels of Bcl-2, affects lymphocytes proliferation and survival (such as Th, B, and NK cells) in patients with cancer.^18,20^

Our results show that Bax mean MFI was higher in all four lymphocyte types in OC patients, but Bcl-2 MFI of all cell types was significantly lower in OC stage IV. This may be explained by the influence of Bax in cancer development, and of Bcl-2 in its progression to stage IV. We also found a high Bax/Bcl-2 ratio in OC stage IV patients. These results corroborate those reported by Kim, et al.^36^ (2004) who found a high Bax/Bcl-2 ratio in circulating CD8^+^ T cells of patients with HNSCC. Tumor size, lymph node involvement, and Bax/Bcl-2 ratio were also higher in advance-stage tumors in our study, which may suggest that Bax/Bcl-2 ratio levels are associated with OSCC aggressiveness. However, further studies with a larger cohort are necessary to clarify this correlation.

The limited number of patients with OC stage I–III poses a limitation for our study. We suggest further studies to be conducted with a larger number of patients to confirm our findings. In conclusion, our results show that lymphocyte apoptosis and Bax/Bcl-2 ratios were higher in patients with OC in stage 4, T4, and N3 tumors, indicating that they play an important role in cancer prognosis. These target molecules may prove useful for supportive treatment in the future.
